# Pathological Assessment of the Appendix in Appendectomies Performed in Children

**DOI:** 10.34172/aim.2024.38

**Published:** 2024-05-01

**Authors:** Farzaneh Javanmard, Yasin Hasanzadegan Sadegh

**Affiliations:** ^1^Department of Pathology, Urmia University of Medical Sciences, Urmia, Iran; ^2^Urmia University of Medical Sciences, Urmia, Iran

**Keywords:** Appendectomy, Children, Histopathology

## Abstract

**Background:**

Acute appendicitis is known as the most common diagnosis of acute abdomen leading to surgery. Therefore, timely diagnosis is of special importance. This study was conducted with the aim of pathological assessment of the appendix in appendectomies performed in children to determine the rate of negative appendectomies and the predictors of negative appendectomy and to evaluate the paraclinical tools used in the diagnosis of acute appendicitis.

**Methods:**

This is a cross-sectional descriptive study. All children who underwent appendectomy at Shahid Motahari Hospital in Urmia from March 2021 to March 2022 were examined. The required data including demographic, paraclinical, and final pathology characteristics were collected and recorded. The investigated cases were classified into positive and negative appendectomy categories for comparison.

**Results:**

Among 234 pathology samples of the appendix, 22 cases were related to accidental appendectomy. In addition, 11.3% of cases were negative appendectomy and 88.7% were positive appendectomy. The age range of 8 to 14 years and male gender were associated with a lower negative appendectomy rate (both *P*<0.001). Inflammatory (49.5%) and gangrenous appendicitis (30.2%) were the most commonly reported histopathologies. Sonography had a sensitivity of 84%, a specificity of 79%, and an overall diagnostic accuracy of 83%.

**Conclusion:**

A relatively significant number of accidental and negative appendectomies are performed. More careful investigation and the use of expectant and medical treatment instead of surgery, especially in females and young children, can be effective in improving diagnostic accuracy and preventing negative appendectomies.

## Introduction

 A common presenting symptom in the emergency department is acute abdominal pain, accounting for 5‒10% of all emergency visits.^1^ Acute abdomen has wide and varied differential diagnoses, the most common of which are acute appendicitis, acute cholecystitis, diverticulitis, and peritonitis. Additionally, more complicated cases such as inflammatory bowel diseases and malignancy, vascular conditions such as abdominal aortic aneurysm and acute mesenteric ischemia, rectus muscle hematoma, gynecological and obstetrical conditions such as ectopic pregnancy, ovarian torsion, and urological conditions such as pyelonephritis and renal colic can be mentioned.^2,3^

 Acute appendicitis is the most common diagnosis of acute abdomen leading to surgery, especially in children, which occurs after the obstruction of the appendiceal lumen and development of inflammation in the appendix. Fecaliths, parasites, tumors, foreign bodies, and bacterial and viral agents have been identified as the underlying causes of appendicitis.^4^ There is no specific gene associated with the occurrence of appendicitis; however, the risk of this disease is about three times higher in people who have a positive family history than other people.^5^

 The incidence of AA has been decreasing at a constant rate since the 1940s. In developed countries, the rate of AA is 5.7‒50 patients per 100 000 individuals per year with a peak between 10 and 30 years of age.^6^ The lifetime risk for AA was reported to be 9% in the USA, 8% in Europe, and 2% in Africa, indicating geographic differences.^7^ On the other hand, there is great variety in terms of the presentation, seriousness of the disease, radiological workup, and surgical management of patients with AA.^8^

 The rate of perforation varies from 16% to 40%, with higher frequency seen in younger patients (40%‒57%) and those older than 50 years (55%‒70%).^9^

 The clinical diagnosis of AA is challenging and needs a combination of clinical, laboratory, and radiological assays. The diagnostic workup could be aided by utilizing clinical scoring systems that include physical examination findings and inflammatory markers.^10,11^ Biomarkers are utilized to supplement patient history and clinical examination, particularly in children, women of reproductive age, and elderly patients, when the diagnosis is troublesome. White blood cell count (WBC), C-reactive protein (CRP), and other novel biomarkers, including procalcitonin, can be used to diagnose AA with high specificity and sensitivity.^12^

 According to a recent meta-analysis, ultrasonography has an overall sensitivity of 86% (95% CI, 83‒88) and a specificity of 81% (95% CI, 78‒84).^13^ Its role as first-line investigation is most prominent in children, who typically have slender musculature, lower abdominal fat, and a greater need for radiation avoidance than adult patients.^7^

 Acute appendicitis is divided into three categories, macroscopically and microscopically: normal appendix, simple appendicitis without perforation, and complicated appendicitis (gangrenous, perforated appendix and appendicitis with abscess formation).

 Appendectomy has been associated with negative appendectomy rates of 15%‒39% in large groups.^7^ Traditionally, a negative appendectomy can be either a grossly and/or a histologically normal appendix with no evidence of acute inflammatory reaction.^14^

 The purpose of this study was to determine the histopathological changes of appendectomy specimens, the rate of negative appendectomy, and the predictors of negative appendectomy and to evaluate the accuracy of paraclinical tools used in the diagnosis of acute appendicitis in children.

## Materials and Methods

 In this cross-sectional and retrospective study, all children who underwent appendectomy at Shahid Motahari Hospital of Urmia were examined from March 2021 to March 2022. Demographic information (age and gender), laboratory information (WBCs and CRP), sonography results (diagnostic, indeterminate, negative), and the pathological characteristics of the samples sent to the pathology center were extracted and recorded in the checklist prepared by the researcher. For comparison, patients were categorized into three age groups: under 4 years, 4 to 7 years, and over 7 years. The recorded CRP levels were considered positive at a cut-off value of one or more. As for the level of white blood cells, 13 000/μL was considered as the cut-off value for leukocytosis. The results of sonography based on the performing operator were divided into three categories for comparison: sonography performed in the morning by the associate professors of radiology present in the hospital, sonography performed in the evening and night by radiologist assistants, and sonography performed in out-of-hospital centers. The reported results of sonography were also classified into two categories: diagnostic (cases where the presence of inflammation or complications in the appendix was clearly mentioned) and non-diagnostic (cases where the presence of inflammation or complications was clearly rejected or any specific opinion was not mentioned). Pathology results were also classified into two categories of positive and negative. Positive cases included three general categories as follows: appendix with acute inflammation (including acute appendicitis, periappendicitis, and early appendicitis), complicated appendix (including gangrenous appendicitis, perforated appendicitis, and appendicitis with abscess formation), and rare cases (including chronic inflammation, vascular congestion, obliterative appendicopathy, and reactive follicular hyperplasia). Negative cases also included pathology reports based on normal or appendix vermicularis.

 Statistical analysis was done using SPSS version 21. For quantitative variables, central and dispersion indices (mean and standard deviation) were utilized. For qualitative variables, frequency and frequency percentage were calculated. According to the results of the Kolmogorov-Smirnov test, the age parameter had a normal distribution; therefore, for comparison of the mean age between two groups with positive and negative appendectomy, an independent *t* test was used. To determine the relationship between qualitative parameters, Fisher’s exact and chi-square tests were utilized with a *P* value of less than 0.05. Sensitivity, specificity, positive predictive value (PPV), negative predictive value (NPV), and diagnostic accuracy of the paraclinical tools were calculated based on standard definitions.

## Results

 A total of 236 appendectomy samples were sent to the pathology department of Shahid Motahari Hospital from March 2021 to March 2022. Among them, 2 samples belonged to patients over 18 years of age (which were excluded), 22 samples belonged to surgical cases with a clinical diagnosis other than appendicitis, and 212 pathological samples belonged to appendectomy cases with presurgical diagnosis of acute appendicitis. The pathology results of appendectomy cases in other surgeries showed that most of these appendectomies were performed in intussusception surgeries (72.7%) and according to the post-surgery pathology reports, the appendix was normal in most cases (68.2%).

 The pathology results of 212 cases of appendectomy performed in children showed that in 188 cases (88.7%), the initial clinical diagnosis was consistent with the histopathological results of the appendix (positive appendectomy) and in 24 cases (11.3%), despite the clinical diagnosis of acute appendicitis, the corresponding pathology was reported to be normal (negative appendectomy). Moreover, inflammatory appendicitis with 105 cases (49.5%), and gangrenous appendicitis with 64 cases (30.2%) were the most common pathologies reported, while perforated appendix was reported in 12 cases (5.7%) (Table 1).

**Table 1:** Frequency of Pathology Results in Appendectomy Cases

**Pathology Result**		**No. (%)**
Non-pathologic*		24 (11.3%)
Pathologic	Inflammatory**	105 (49.5%)
	Gangrenous	64 (30.2%)
	Perforated	12 (5.7%)
	Rare cases***	7 (3.3%)

*Normal appendix/appendix vermicularis. **Early appendicitis/acute appendicitis/periappendicitis. ***Obliterative appendicopathy/vascular congestion/reactive follicular hyperplasia.

 The mean age of the children was 8.04 ± 3.07 (age range: 1 to 14 years).

 According to the results of the independent *t*-test, the mean age of children with positive appendectomy (8.1 ± 3.02) was higher compared to those with negative appendectomy (7.2 ± 2.98) (*P* < 0.001). In children under 4 years of age, 20% of appendectomy cases had normal histopathology (negative appendectomy), while only 10% of appendectomy cases in children over 7 years had normal histology (*P*< 0.001) (Table 2).

**Table 2:** Mean Age of Children Based on the Type of Pathology

**Age (y)**	**Appendectomy**		***P *** **value**
	**Positive**	**Negative**	
< 4	20 (80%)	5 (20%)	< 0.001*
4‒7	51 (89.5%)	6 (10.5%)	
> 7	117 (90%)	13 (10%)	
Mean ± SD	8.1 ± 3.02	7.2 ± 2.98	< 0.001**

*Independent t-test; ** Chi-square test.

 In general, among the investigated cases, 118 cases were boys (55.7%) and 94 cases (44.3%) were girls. In male children, 8.5% of appendectomy cases were associated with normal histopathology (negative appendectomy), while in female children, this rate was 14.9%. According to the results of Fisher’s exact test, this difference was significant (*P*< 0.001, OR = 1.89) (Table 3).

**Table 3:** Gender of Appendectomized Children Based on the Type of Pathology

**Gender**	**Appendectomy**		***P *** **Value**
	**Positive**	**Negative**	
Male	108 (91.5%)	10 (8.5%)	< 0.001*
Female	80 (85.1 %)	14 (14.9%)	

*Fisher's exact test.

 The rate of negative appendectomy in cases with negative or trace CRP level (23%) was significantly higher than that in cases with triple positive CRP level (2.4%) (*P*< 0.001). Considering the diagnostic cut-off point of one positive and more, the test had a sensitivity of 70% and a specificity of 71% (Table 4).

**Table 4:** CRP Results Based on the Type of Pathology

**CRP**	**Appendectomy**		***P *** **Value**
	**Positive**	**Negative**	
Negative or trace	57 (87%)	17 (23%)	< 0.001*
One or two plus	91 (83.8%)	6 (16.2%)	
Three plus	40 (97.6%)	1 (2.4%)	

*Chi-square.

 The mean white blood cell count in children with positive pathology results (11376.06 ± 3771.02/μL) was significantly higher than the level of white blood cells in children with negative pathology (10116.01 ± 2299/μL) (*P*= 0.026). Therefore, considering the level of 13000/μL as the cut-off value for leukocytosis, the prevalence of leukocytosis was significantly lower in children with negative appendectomy (13% to 6%, *P*< 0.001). The sensitivity and specificity of leukocytosis were 25 and 87%, respectively (Table 5).

**Table 5:** WBC Count Based on the Type of Pathology

**Leukocytosis **	**Appendectomy**		***P *** **Value**
	**Positive**	**Negative**	
Positive	47 (94%)	3 (6%)	< 0.026*
Negative	141 (87%)	21 (13%)	
WBC/μL, Mean ± SD	11376.06 ( ± 3771.02)	10116.01 ( ± 2299.1)	< 0.001**

*Independent *t* test; ** Fisher's exact test.

 The results of sonography before appendectomy in appendectomized children showed that only 5 cases (3.1%) had a normal pathology report among the cases with diagnostic and positive sonography, while 38.8% of the cases with non-diagnostic and negative sonography had a final normal pathology report (*P*< 0.001). The sensitivity and specificity of sonography as a diagnostic method before surgery were 84% and 79%, respectively. Moreover, the PPV, NPV, and accuracy of this method were 97%, 39% and 83%, respectively (Table 6).

**Table 6:** Sonography Results Based on the Type of Pathology

**Sonography**	**Appendectomy**		***P *** **Value**
	**Positive**	**Negative**	
Positive	158 (96.9%)	5 (3.1%)	< 0.001
Negative/indeterminate	30 (61.2%)	19 (38.8%)	

*Fisher's exact test.

 In Table 7, the results of sonography before appendectomy are shown according to the operator who performed it. Most of the sonography examinations had been done by university professors present in the hospital, with 90 and 79% sensitivity and specificity, respectively.

**Table 7:** Sonography Results Based on the Operator and Pathology Type

**Operator**	**Sonography**	**Appendectomy**		**Sensitivity**	**Specificity**
		**Positive**	**Negative**		
Professor	Positive	110	4	90%	79%
	Negative/indeterminate	12	15		
Resident	Positive	28	0	70%	100%
	Negative/indeterminate	12	3		
Out-of-hospital report	Positive	20	1	77%	50%
	Negative/indeterminate	6	1		

## Discussion

 Appendicitis is a very common problem in the abdomen that often requires surgery; appendectomies are performed more often than any other surgery in the world.^15^ The decision to send appendix samples for routine examination depends on the clinician’s preference.^16^ Some authors disagree with this policy and believe that appendices should be sent for examination only if a clear abnormality is seen during surgery.^17,18^ They support their opinion by saying that abnormal results are not common, and it costs a lot to process samples. Others believe that examining the appendix under a microscope is important to make sure the diagnosis of acute appendicitis is correct.^19^ This examination can also find other issues that may not be obvious during the operation and could affect the way the patient is subsequently treated.

 In Shahid Motahari Hospital of Urmia, as children’s treatment center, all cases related to appendectomy of children are investigated from the histopathological point of view. In addition, some surgeons often remove the appendix incidentally during various types of surgery involving the reproductive organs, as well as urinary and digestive systems.^20^ Based on this study, out of 234 appendectomies, 9.4% were accidental appendectomies in surgeries such as intussusception, and most of them had normal histopathology. A review with findings similar to ours showed that about 10%‒20% of accidental appendectomy cases in children had normal histopathology.^21^ On the other hand, Wang et al found that the mean length of stay in the hospital, cost, and rate of related complications in cases of intussusception surgery with accidental appendectomy were significantly higher than in cases without an accidental appendectomy.^22^ According to these results, the necessity of random appendectomy is still discussed. However, incidental appendectomy should not be performed in patients whose conditions are unstable, patients diagnosed with Crohn’s disease, patients with an inaccessible appendix, patients undergoing radiation treatment, patients who are immunosuppressed, and patients with vascular grafts or foreign material.^23^

 Based on the findings of this study, 88.7% of the appendectomy cases were accompanied by an abnormal pathological report (positive appendectomy) and 11.3% were negative appendectomies. In 2018, Zouari et al found that 11.2% of children in Tunisia had a negative appendectomy, which is in line with the present study,^24^ while Monajemzadeh et al reported a slightly higher rate of negative appendectomy in children compared to the present study (20%).^25^ The findings of the studies conducted in populations consisting of children and adults, such as the study by Amanollahi et al and Erfani Fam et al, also indicated a frequency of 10 to 15% for negative appendectomies.^26,27^ In the study by Yazar et al, the prevalence of negative appendectomies in the adult population was reported to be 11%.^28^ Another study conducted in 2016 in China stated that the rate of negative appendectomies in the adult population was 25%,^29^ while Khorshidi et al in Hamedan reported that the rate of negative appendectomies in adult patients was about 70%.^30^ The general review of the results of different studies and the results of the present study show that there is a diversity in the frequency of unnecessary appendectomies in different centers, which is related to the study year and the study population (children, adults, or a combination of both). More recent studies,^31,32^ compared to older studies,^30^ have reported a lower rate of negative appendectomies, which can be due to the increase in the diagnostic accuracy of paraclinical methods as well as the increase in the use of expectant treatment in cases of suspected appendicitis. In this regard, the results of the systematic review also indicated a significant decrease in negative appendectomies during the period from 1998 to 2008.^33^ On the other hand, the rate of negative appendectomies in adults is slightly higher than in children. In this regard, Lee and Ho reported a much higher rate of unnecessary appendix surgeries in adults compared to children.^34^ However, some studies with a small sample size have had contradictory results.^35^

 Based on the results of our study, inflammatory appendicitis was reported in about half of the histopathological results, followed by gangrenous and perforated appendicitis. Monjemizadeh et al reported inflammatory-suppurative appendicitis as the most common histopathology of appendectomy cases.^25^ In India, Sujatha et al reported that the total frequency of acute inflammatory suppurative appendicitis was about 50%; however, unlike the present study, cases of perforated appendicitis (1%) and gangrenous appendicitis (2.2%) were much less frequent.^36^ A study in Saudi Arabia and another study in Pakistan stated that the most common histopathological findings in appendectomy cases were inflammatory and suppurative, but unlike the present study, the frequency of gangrene and perforation was lower.^37,38^ The above-mentioned results show that acute suppurative appendicitis is the most common cause of appendectomy; however, unlike most similar studies, the number of cases of perforated and gangrenous appendicitis in this study was higher, which could be due to the late diagnosis of the cases admitted to this center.

 In the present study, two histopathological cases related to the closure of the appendix lumen (obliterative appendicopathy) were also reported; this histopathology occurs after the removal of the mucosal and submucosal layers of the appendiceal lumen and its replacement with fibrous tissue, which is often known as a part of the aging process, and most of the reported cases occurred in elderly people,^39^ Nevertheless, some studies have mentioned it in the form of case reports in children.^40^

 In our research, most of the children who had appendectomy surgery were between 8 and 14 years old, with an average age of 8 years. The frequency of male children was higher compared to female children. Moreover, older age, especially between 8 and 14 years, and male gender have been associated with a lower rate of negative appendectomy. In a study conducted in Oman, in line with this study, there were more boys than girls who had their appendix removed, and also the children under 12 years of age had a higher rate of unnecessary appendix removal compared to adults over 12 years.^35^ In the study by Zouari et al, the average age of children who underwent appendectomy was reported to be 9 years. However, contrary to the current study, the rate of unnecessary appendix removal did not show a significant correlation with the gender of the children.^24^ In a study by Malekpour et al on all appendectomy cases during 5 years in a medical center in Tehran, fewer men than women had unnecessary appendix removal surgeries, which is in line with the present study.^41^ In a study by Kadi et al, more appendectomies were performed in men and significantly, more women had unnecessary appendix surgeries, which confirms the results of the present study.^42^ Other similar studies have emphasized the higher frequency of negative appendectomy cases in women than in men in all age groups.^33,38^ This difference can be justified based on the anatomical structure of the female body; in other words, the presence of structures such as the ovary and uterus leads to an increase in the number of differential diagnoses mimicking acute appendicitis in women. On the other hand, children under the age of 4 are more likely to have unnecessary appendix surgeries because it is difficult for doctors to determine whether the appendix is actually the problem. This is because it is difficult to ask the child questions about their symptoms and perform a thorough examination.

 Based on the results of this study, when the CRP level is one or higher, it is 70% likely to correctly identify the problem, and it is 71% likely to correctly rule out the problem. On the other hand, when there is a high level of white blood cells (13 000/μL), it is only 25% likely to correctly identify the problem, but it is 87% likely to correctly rule out the problem. Besides, sonography with 84% sensitivity and 83% diagnostic accuracy has been able to help the surgeon in the accurate diagnosis of appendicitis. On the other hand, sonography evaluations performed by university professors in the morning time have higher sensitivity and specificity than those performed by assistants. In various studies, it has been shown that the diagnostic accuracy of the CRP level is higher compared to leukocytosis, especially in cases of perforation.^27,28,43^ Nevertheless, the final diagnosis is more accurate with a combination of the patient’s clinical condition and various laboratory parameters. Regarding sonography, the research conducted by Amanollahi et al found that the sensitivity and specificity were 98% and 76%, which is similar to our study,^26^ while some other studies have reported sensitivity and specificity of less than 60% for sonography.^27,36^ The differences in the diagnostic accuracy of sonography can be explained in terms of the differences in the operator’s skill and the level of cooperation of the patients.

## Conclusion

 Inflammatory, gangrenous, and perforated appendicitis are the most common pathologies leading to appendectomy in children; nevertheless, a relatively significant number of accidental and negative appendectomies are also performed. Male gender and older age in children were associated with a lower rate of negative appendectomy; therefore, a more careful investigation and the use of expectant and medical treatment instead of surgery, especially in females and young children, can be effective in improving diagnostic accuracy and preventing negative appendectomies.

 Paraclinical tests alone have low diagnostic accuracy for acute appendicitis, and considering their results together with examinations and history of patients can be helpful.

 Sonography, as the most commonly used diagnostic method, can be associated with good diagnostic accuracy, especially in cases where it is done electively in the morning by radiology professors.
